# Anterior Uveitis Secondary to Avelumab and Pembrolizumab in a Patient with Metastatic Renal Cell Carcinoma—A Case Report

**DOI:** 10.3390/reports7010019

**Published:** 2024-03-06

**Authors:** Wei-Lun Chu, Kai-Chun Cheng, Pei-Kang Liu, Hung-Chi Lai, Kuo-Jen Chen, Yo-Chen Chang

**Affiliations:** 1 Department of General Medicine, Kaohsiung Medical University Hospital, Kaohsiung 807, Taiwan; t5368836@gmail.com; 2 Department of Ophthalmology, Kaohsiung Municipal Siaogang Hospital, Kaohsiung 812, Taiwan; pington64@kmu.edu.tw (K.-C.C.); pkliu@kmu.edu.tw (P.-K.L.); kjchen.oph@gmail.com (K.-J.C.); 3 Department of Ophthalmology, Kaohsiung Medical University Hospital, Kaohsiung 807, Taiwan; phillip7678@gmail.com; 4 Department of Ophthalmology, School of Medicine, College of Medicine, Kaohsiung Medical University, Kaohsiung 807, Taiwan; 5 Graduate Institute of Medicine, College of Medicine, Kaohsiung Medical University, Kaohsiung 807, Taiwan

**Keywords:** case report, uveitis, immune checkpoint inhibitor, avelumab, pembrolizumab

## Abstract

We present an unusual case of uveitis secondary to avelumab and pembrolizumab in a 39-year-old Taiwanese male with stage IV clear cell renal cell carcinoma (ccRCC) and lung metastasis, who initially received pembrolizumab as his primary treatment. However, the patient experienced skin and liver immune-related adverse events (irAEs) after the seventh dose of pembrolizumab, which prompted a switch to avelumab. The patient began to experience gradual blurring of vision after completing the fifth cycle of avelumab immunotherapy. Ophthalmic examinations revealed findings consistent with bilateral anterior uveitis. Despite an initial lack of significant improvement with steroid treatment, the patient’s vision and inflammation improved upon discontinuation of avelumab. Due to the occurrence of uveitis, avelumab was switched back to pembrolizumab. However, three months after initiating pembrolizumab, the patient developed foggy vision and bilateral anterior uveitis with cystoid macular edema (CME). The administration of topical, oral, and subconjunctival steroids resulted in an improvement in vision and the resolution of CME, without the need to discontinue pembrolizumab. Over the subsequent eighteen months, there has been no recurrence of uveitis, and there is no evidence of relapse or further metastasis in his ccRCC.

## 1. Introduction

Immune checkpoint inhibitors (ICIs) are monoclonal antibodies that have revolutionized the treatment landscape of various cancers by targeting cytotoxic T-lymphocyte-associated antigen-4 (CTLA-4), programmed cell death protein-1 (PD-1), and programmed death-ligand 1 (PD-L1) [1]. PD-1 is a receptor expressed on activated immune cells, and it downregulates immune cell activity when bound by programmed death-ligand 1 (PD-L1). PD-L1 is found to be expressed on cancer cells, enabling tumors to induce anergy or apoptosis in immune cells specific to the tumor. This pathway facilitates certain malignancies in evading the immune system [2,3]. Monoclonal antibodies such as pembrolizumab and nivolumab bind to PD-1 on immune cells, while antibodies like atezolizumab, avelumab, and durvalumab bind to PD-L1 on tumors. These medications effectively block PD-1/PD-L1 interactions, preventing immune cell deactivation in the tumor microenvironment [4].

While the pathways targeted by ICIs also regulate T-cell interactions with non-tumor cells and enhance the natural immune response, it is not surprising that a considerable incidence of systemic irAEs has been observed following exposure to ICIs [5]. These adverse effects span various organ systems, encompassing the skin, lungs, liver, kidneys, heart, bone marrow, and gastrointestinal system. Common side-effects include skin rash, pruritus, diarrhea, lymphopenia, and elevated liver function markers [6]. Furthermore, ocular and orbital side-effects, including conjunctivitis, episcleritis, scleritis, uveitis, keratitis, corneal graft rejection, and dry eye syndrome, are frequently associated with irAEs [7,8]. Among these, uveitis is estimated to occur in around 1% of patients undergoing ICI therapy and this irAE characterized by uveal tract inflammation can have a severe impact on vision if left untreated.

The onset of ICIs side-effects may manifest weeks to months after the initiation of therapy. Typically, these effects can be effectively managed using topical or systemic steroids without the discontinuation of the medication. Nevertheless, in advanced cases, the suspension of therapy for several months or even permanent discontinuation may become imperative [9,10]. Research indicates that a majority of patients developing irAEs exhibit preceding tumor regression or retardation. Given that the risk of irAE development tends to escalate with the ICI dose, employing individualized dosing could potentially empower patients to sustain therapeutic efficacy while minimizing toxicity [11].

To the best of our knowledge, uveitis secondary to avelumab has only been mentioned by the U.S. Food and Drug Administration (FDA) but has not been previously reported in a well-documented case report. Additionally, we reported a rare occurrence of uveitis with different ICIs. In this case report, we describe a 39-year-old male with stage IV ccRCC who experienced bilateral anterior uveitis following avelumab treatment, which improved upon discontinuing avelumab. However, he later had a recurrence of bilateral anterior uveitis while receiving pembrolizumab therapy.

## 2. Detailed Case Description

A 39-year-old Taiwanese male with stage IV ccRCC and metastasis to the bilateral lungs, his status post-left radical nephrectomy, was receiving immunotherapy with intravenous pembrolizumab 100 mg every three weeks. He experienced irAEs, including elevated liver enzymes and hand–foot syndrome, after the seventh dose of pembrolizumab. As a result, pembrolizumab was replaced with intravenous avelumab 800 mg every three weeks. However, after the fifth dose of avelumab, the patient began to notice a gradual blurring of vision in both eyes.

**Figure 1:**
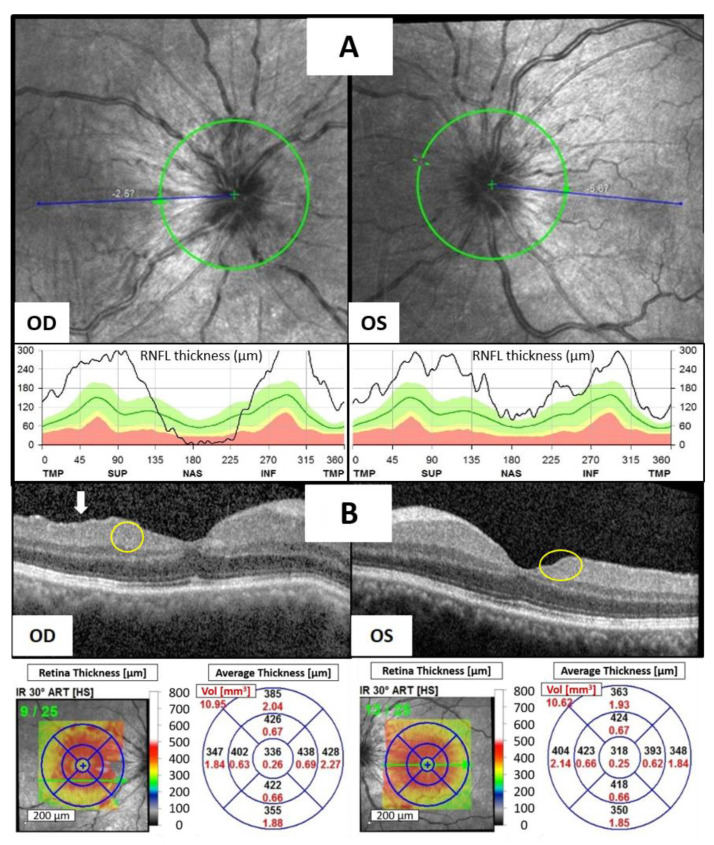

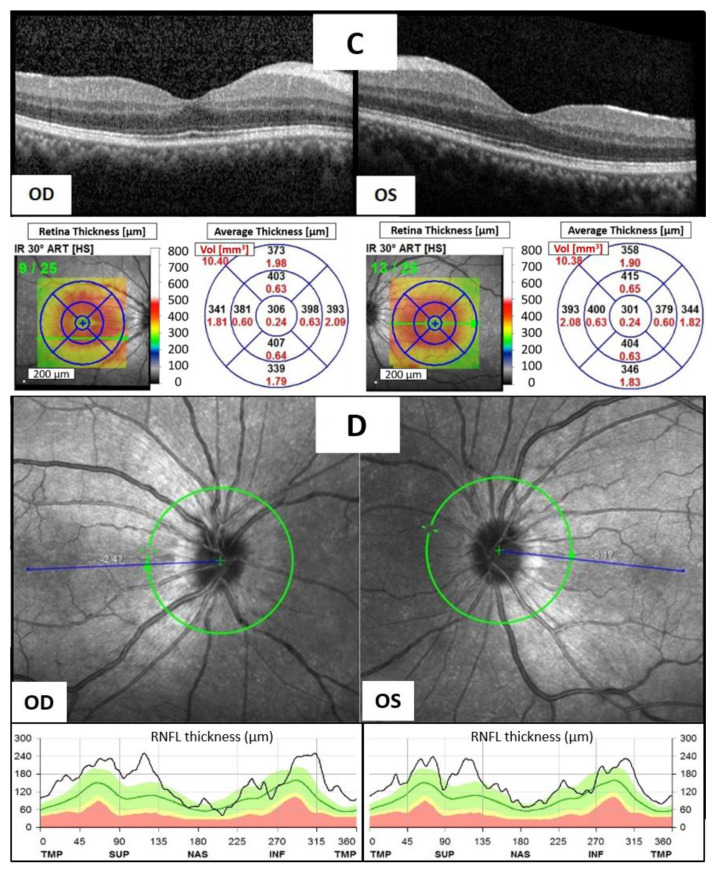
Avelumab-induced uveitis on OCT of the right eye (OD) and the left eye (OS). (**A**) OCT and retinal nerve fiber layer (RNFL) thickness (black line) showed prominent bilateral disc edema after the fifth dose of avelumab administered. (**B**) Multiple HRF (yellow circles) in both eyes, DRIL (white arrow) in right eye and increased inner retina thickness of macula in both eyes were noted after the fifth dose of avelumab administered. (**C**) Less inner retina thickening, resolution of HRF and DRIL of bilateral macula after discontinuing avelumab for ten days. (**D**) OCT and RNFL thickness (black line) showed significant resolution of bilateral disc edema after discontinuing avelumab for 32 days.

At the time of assessment, the best corrected visual acuity (BCVA) was measured at 20/70 in the right eye and 20/40 in the left eye. The intraocular pressure was 12.3 mmHg in the right eye and 9.4 mmHg in the left eye. Slit-lamp examination revealed significant ciliary injection, fine keratic precipitates with 2+ cells, and 1+ flare in the anterior chamber (AC) of both eyes. Neither iris nodules nor peripheral anterior synechia were noted in both his eyes. Indirect ophthalmoscopy revealed bilateral disc edema without evident snow balls, retinal vasculitis, retinal nodules, or choroidal nodules. Optical coherence tomography (OCT) showed marked bilateral optic disc edema (Figure 1A), multiple hyperreflective foci (HRF, yellow circles) in both eyes, disorganization of the retinal inner layers (DRIL, white arrow) in the right eye, and an increased inner retina thickness of macula in both eyes (Figure 1B). Laboratory investigations for infectious and inflammatory causes including complete blood count, differential count, serum calcium, gamma-globulin, KL-6, immunoglobulin G/A/M, complement C3/C4, antinuclear antibody, Venereal Disease Research Laboratory (VDRL), Treponema pallidum hemagglutination assay (TPHA), antibody for Human Immunodeficiency Virus (HIV), blood culture, and urine culture yielded no significant findings. Chest computed tomography showed no evidence of hilar lymphadenopathy, indicating a low likelihood of sarcoidosis. The patient had no history of rheumatoid disease or uveitis, and there was no evidence of metastatic cancer in either eye.

Given the suspicion of avelumab-induced bilateral anterior uveitis, the patient was given topical 1% prednisolone acetate suspension four times daily and oral prednisolone 10 mg per day. However, his condition showed only minimal improvement after four weeks of steroid treatment, leading to the decision to discontinue avelumab while maintaining the steroid therapy. Ten days later, BCVA improved to 20/30 in the right eye and 20/25 in the left eye. AC inflammation had decreased to only a trace number of cells in both eyes, and OCT showed less inner retina thickening of bilateral macula and a marked resolution of HRF and DRIL (Figure 1C). A period of 32 days later, the patient’s vision had returned to 20/20 in both eyes, and no ocular inflammation was observed. OCT revealed complete resolution of bilateral disc edema (Figure 1D). The significant improvement following the discontinuation of avelumab strongly suggested a diagnosis of avelumab-induced uveitis.

**Figure 2:**
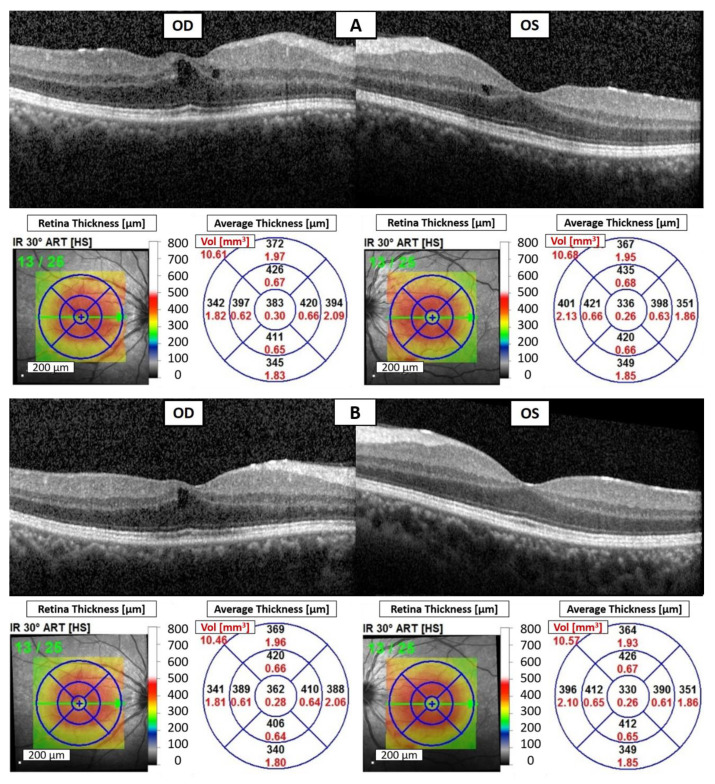

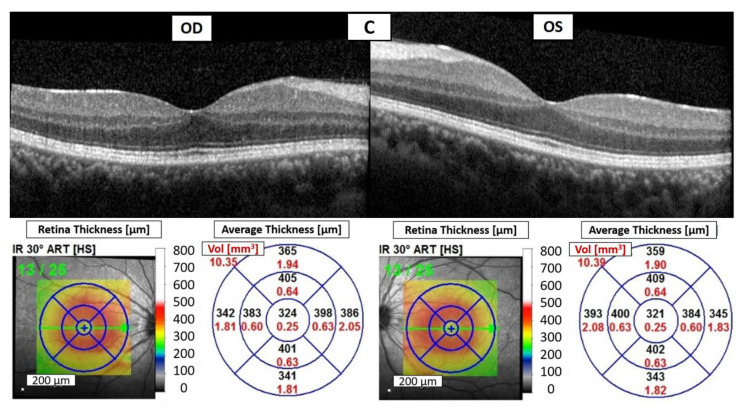
Pembrolizumab-induced uveitis on OCT of the right eye (OD) and the left eye (OS). (**A**) Bilateral CME after three months of pembrolizumab administered. (**B**) Decreased CME of both eyes after steroid treatment for 14 days. (**C**) Total resolution of bilateral CME after steroid treatment for 35 days.

Due to avelumab-induced uveitis, avelumab was discontinued. Given the limited options, the ICI was switched back to intravenous pembrolizumab at a dosage of 100 mg every three weeks to manage his underlying disease. Unfortunately, the patient experienced foggy vision in both eyes again after the fourth dose of pembrolizumab treatment. The BCVA was measured at 20/30 in both eyes. Slit-lamp examination revealed fine keratic precipitates, 3+ cells, and 1+ flare in the AC of both eyes. OCT demonstrated bilateral CME (Figure 2A). Although blurred vision had not been noted previously during the first period of pembrolizumab treatment, a diagnosis of pembrolizumab-induced bilateral anterior uveitis was still favored due to the absence of significant findings in the laboratory workup for infectious and inflammatory etiologies, as well as metastatic cancer in both eyes.

Despite the suspicion of pembrolizumab-induced bilateral anterior uveitis, pembrolizumab continues to be administered to manage the patient’s ccRCC. The treatment plan includes the use of topical 1% prednisolone acetate suspension four times daily, oral prednisolone at a dosage of 10 mg per day, and a subconjunctival injection of 2 mg of betamethasone once every two weeks. A total of three injections were administered throughout the treatment period. A period of 14 days later, BCVA improved to 20/25 in both eyes, and the cell count in AC decreased to 2+ without flare in both eyes. OCT of both eyes demonstrated a reduction in CME (Figure 2B). A period of 35 days later, there was no AC cell or flare in both eyes, and OCT showed significant resolution of bilateral CME (Figure 2C). The improvement strongly supported a diagnosis of pembrolizumab-induced uveitis. Over the following eighteen months, the patient continued to receive topical 0.1% fluorometholone four times daily and oral prednisolone 5 mg per day, and there were no recurrences of uveitis during regular pembrolizumab treatment. Additionally, the patient’s stage IV ccRCC remained controlled with regular pembrolizumab usage, and no new distant metastases occurred.

## 3. Discussion

ICIs have become a standard treatment for various cancers. Consequently, a corresponding increase in ophthalmological irAEs is expected, and it is crucial for ophthalmologists to develop strategies for managing these ocular side-effects.

This case highlights a patient who experienced two distinct episodes of uveitis, each associated with a different ICI. The first episode of uveitis in the patient manifested as bilateral avelumab-induced anterior uveitis, classified as Grade 3 according to the Common Terminology Criteria for Adverse Events (CTCAE). In the second episode of uveitis, the patient exhibited bilateral pembrolizumab-induced anterior uveitis, which was also classified as Grade 3 by CTCAE.

Due to the fact that the patient had previously received pembrolizumab seven times and avelumab five times before the onset of the first uveitis, it raises a question of whether the first occurrence of uveitis was caused by avelumab or pembrolizumab. According to previous literature statistics [12], uveitis induced by pembrolizumab has an average onset time of approximately 84 days, while uveitis induced by PD-L1 inhibitors has an average onset time of around 25 days, meaning uveitis induced by PD-1 inhibitors tends to have a longer onset time than that induced by PD-L1 inhibitors. Additionally, the recovery time of uveitis after treatment is typically around 3 to 4 weeks regardless of the type of ICI used.

In this case, we favor attributing the patient’s uveitis to avelumab for several reasons. First, the patient experienced the first episode of uveitis 323 days after the initial use of pembrolizumab, which exceeds the average onset time observed in the literature. Second, the improvement in ocular inflammation after discontinuing avelumab supports the diagnosis of avelumab-induced uveitis. This perspective is further substantiated by the fact that the pembrolizumab-induced uveitis that subsequently occurred in the patient could indeed be controlled with steroids. The resolution time from uveitis after discontinuing avelumab was 32 days, which is also consistent with previous research statistics. The temporal association between avelumab administration and the onset of uveitis, along with the resolution of ocular inflammation after discontinuation of the drug, further supports the diagnosis.

Recent research suggests that emerging anatomic biomarkers in OCT, including DRIL, HRF, intraretinal (IR) cysts, and the integrity of the ellipsoid zone (EZ), hold promise for enhancing our comprehension of individual therapy response, disease progression, and refining treatment strategies [13]. Grewal et al. demonstrated a robust correlation between DRIL, HRF, IR cysts, and disruption of the EZ with visual acuity in individuals affected by current or resolved uveitic CME [14]. In our case, during the episode of avelumab-induced uveitis, we observed multiple HRF in the inner layers of both eyes, DRIL in the right eye, and an increased thickness of the inner retina in the macula of both eyes. Additionally, during the episode of pembrolizumab-induced uveitis, CME was noted in both eyes. These findings may have contributed to the patient’s blurred vision. Subsequently, as the uveitis improved, the regression of HRF, DRIL, and CME accompanied the improvement in visual acuity, providing additional support for this observation.

The pathophysiology of ICI-induced uveitis is not completely understood, but it is currently thought to stem from alterations in the functionality of the body’s immune system. This primarily involves the disruption of autoimmune tolerance or an increased sensitivity to antigen recognition, leading to autoimmune inflammation [6,15]. In addition to the more commonly studied cytotoxic T-cell, treatment with ICIs tends to shift T cells more towards T helper cell 1 or T helper cell 17-driven inflammation and autoimmune responses [16]. This phenomenon may be a contributing factor to the observed anterior uveitis. Moreover, some patients may have a predisposition to developing irAEs, including uveitis, due to genetic factors or pre-existing autoimmune conditions [15,16]. In this case, the patient experienced a second episode of uveitis after discontinuing avelumab and switching back to pembrolizumab. We speculate that uveitis previously induced by avelumab caused immune dysregulation, making the patient predisposed to developing irAEs even when switching back to pembrolizumab, despite the differences in their mechanisms (PD-1 inhibitor and PD-L1 inhibitor). This resulted in the occurrence of uveitis 84 days after switching back to pembrolizumab, which did not occur during the patient’s first course of using pembrolizumab for approximately six months.

The development of uveitis with two different ICIs in the same patient is a rare occurrence, and it raises questions about the optimal management strategy for patients who experience irAEs with multiple ICIs. In general, efforts should be made to continue the use of ICI whenever possible to improve the patient’s chances of survival. In our case, the anterior uveitis induced by avelumab, which demonstrated relative resistance to topical and oral corticosteroid administration, greatly improved upon discontinuation of avelumab. In contrast, the anterior uveitis induced by pembrolizumab was resolved with corticosteroid administration. This aligns with the literature suggesting that pembrolizumab-induced uveitis can be effectively managed with corticosteroids in the majority of cases [12]. However, we did not utilize subconjunctival steroid injection for treating avelumab-induced uveitis. Therefore, we cannot determine whether using subconjunctival steroid injection would yield better therapeutic outcomes. The decision to discontinue avelumab was chosen at that time as a potentially quicker way to improve the patient’s vision, considering the relatively short half-life of Avelumab. Early recognition and prompt management of irAEs are crucial to minimize the risk of complications [17]. Nevertheless, further research is needed to understand the risk factors for ICI-induced uveitis and to establish the most effective treatment strategies for these patients.

## 4. Conclusions

In conclusion, this report adds to the growing pool of data characterizing uveitis that ICIs such as avelumab and pembrolizumab are able to cause. As one of the few cases documenting avelumab-induced uveitis, our findings indicate that it shares similar characteristics with other PD-L1 inhibitors and can be effectively treated with corticosteroids and with ICI discontinuation. However, while avelumab discontinuation was deemed necessary in this case due to prolonged inflammation, it may not be universally required. This case report also presents a rare occurrence of uveitis associated with two different ICIs, avelumab and pembrolizumab. Once a patient develops uveitis secondary to ICI, based on the patient’s presentation, it is possible that other ICIs may also induce uveitis. The development of uveitis with two different ICIs highlights the importance of the careful monitoring of patients receiving ICI therapy for potential irAEs, including ocular manifestations. Early recognition and appropriate management of ICI-induced uveitis are crucial to prevent complications and preserve vision. Further studies are needed to better understand the pathophysiology of ICI-induced uveitis and to develop more effective strategies for managing patients who experience irAEs with multiple ICIs.

## Data Availability

The data presented in this study are available on request from the corresponding author. The data are not publicly available, due to privacy.
